# Impact of corrosion inhibitors on antibiotic resistance, metal resistance, and microbial communities in drinking water

**DOI:** 10.1128/msphere.00307-23

**Published:** 2023-09-08

**Authors:** Lee K. Kimbell, Emily Lou LaMartina, Stan Kohls, Yin Wang, Ryan J. Newton, Patrick J. McNamara

**Affiliations:** 1 Department of Civil, Construction and Environmental Engineering, Marquette University, Milwaukee, Wisconsin, USA; 2 School of Freshwater Sciences, University of Wisconsin-Milwaukee, Milwaukee, Wisconsin, USA; 3 Department of Civil and Environmental Engineering, University of Wisconsin-Milwaukee, Milwaukee, Wisconsin, USA; Antimicrobial Development Specialists, LLC, Nyack, New York, USA

**Keywords:** antimicrobial resistance, metals, zinc, sodium orthophosphate, pollution control

## Abstract

Corrosion inhibitors, including zinc orthophosphate, sodium orthophosphate, and sodium silicate, are commonly used to prevent the corrosion of drinking water infrastructure. Metals such as zinc are known stressors for antibiotic resistance selection, and phosphates can increase microbial growth in drinking water distribution systems (DWDS). Yet, the influence of corrosion inhibitor type on antimicrobial resistance in DWDS is unknown. Here, we show that sodium silicates can decrease antibiotic resistant bacteria (ARB) and antibiotic-resistance genes (ARGs), while zinc orthophosphate increases ARB and ARGs in source water microbial communities. Based on controlled bench-scale studies, zinc orthophosphate addition significantly increased the abundance of ARB resistant to ciprofloxacin, sulfonamides, trimethoprim, and vancomycin, as well as the genes *sul1*, *qac*EΔ1, an indication of resistance to quaternary ammonium compounds, and the integron-integrase gene *intI*1. In contrast, sodium silicate dosage at 10 mg/L resulted in decreased bacterial growth and antibiotic resistance selection compared to the other corrosion inhibitor additions. Source water collected from the drinking water treatment plant intake pipe resulted in less significant changes in ARB and ARG abundance due to corrosion inhibitor addition compared to source water collected from the pier at the recreational beach. In tandem with the antibiotic resistance shifts, significant microbial community composition changes also occurred. Overall, the corrosion inhibitor sodium silicate resulted in the least selection for antibiotic resistance, which suggests it is the preferred corrosion inhibitor option for minimizing antibiotic resistance proliferation in DWDS. However, the selection of an appropriate corrosion inhibitor must also be appropriate for the water chemistry of the system (e.g., pH, alkalinity) to minimize metal leaching first and foremost and to adhere to the lead and copper rule.

**IMPORTANCE:**

Antibiotic resistance is a growing public health concern across the globe and was recently labeled the silent pandemic. Scientists aim to identify the source of antibiotic resistance and control points to mitigate the spread of antibiotic resistance. Drinking water is a direct exposure route to humans and contains antibiotic-resistant bacteria and associated resistance genes. Corrosion inhibitors are added to prevent metallic pipes in distribution systems from corroding, and the type of corrosion inhibitor selected could also have implications on antibiotic resistance. Indeed, we found that sodium silicate can minimize selection of antibiotic resistance while phosphate-based corrosion inhibitors can promote antibiotic resistance. These findings indicate that sodium silicate is a preferred corrosion inhibitor choice for mitigation of antibiotic resistance.

## INTRODUCTION

The proliferation of antibiotic resistance in natural and engineered environments is a serious threat to human health (1). Bacteria become resistant to antibiotics through genetic mutations or by acquiring antibiotic resistance genes (ARGs) from their surrounding environment (2). Aquatic ecosystems harbor a vast pool of ARGs that can undergo mutations, recombination, and selection events (3). ARGs are considered emerging contaminants in aquatic ecosystems (4), and the occurrence of ARGs in drinking water is a potential risk to human health (5). Groundwater and surface waters such as lakes and rivers are often used as source waters to drinking water treatment plants and are considered important reservoirs for antibiotic resistant bacteria (ARB) and associated ARGs (6 – 8). Drinking water treatment processes do not completely remove ARB and ARGs, which are subsequently transported through drinking water distribution systems (DWDS) (9, 10). Drinking water disinfection processes use chlorine or chloramines to inhibit microbial growth in distribution systems; however, exposure to chlorine and metals at subinhibitory concentrations can also select for antibiotic resistance in aquatic environments such as DWDS (11 – 13).

The Lead and Copper Rule (LCR) was introduced by the US Environmental Protection Agency in 1991, which requires drinking water utilities to perform optimized corrosion control treatment to reduce dissolved copper and lead levels in drinking water (14). Drinking water utilities have three options for corrosion control treatment: (i) pH adjustment, (ii) maintenance of a disinfectant residual to develop Pb(IV) scale, and (iii) using corrosion inhibitors such as orthophosphates and sodium silicates (15, 16). The most common method of corrosion control in the U.S. is the use of phosphate-based corrosion inhibitors, including orthophosphates and polyphosphates, with 56% of systems reporting orthophosphate dosages from 1 to 3 mg/L (17, 18). Consideration of a corrosion control agent can be based on water chemistry (e.g., pH, alkalinity), and typically silicate- and phosphate-based agents work in different ranges. Corrosion inhibitors containing phosphate may serve as a nutrient source for bacterial growth and increase microbial community diversity in DWDS (19, 20). Indeed, a recent study demonstrated that zinc orthophosphate addition to drinking water can select for increased absolute and relative abundance of ARB and ARGs compared to untreated controls (21). The addition of zinc orthophosphate increased total bacterial abundance (i.e., 16S rRNA gene copies), as well as total and relative ARB and ARG abundance, indicating that the observed changes were not due to increased bacterial abundance alone.

Sodium silicates have the general formula of Na_2_O•xSiO_2_, where “x” varies from 1.6 to 3.22 for silicates commonly used for drinking water treatment (16, 22). Silicates are an alternative to phosphate-based inhibitors for corrosion control in DWDS and have been reported as an effective treatment for dissolved lead control under some conditions (23, 24). Studies have reported sodium silicate dosages ranging from 10 to 48 mg/L as an inferior corrosion control treatment compared to 1 mg/L orthophosphate treatment in circumneutral pH water with low to moderate alkalinity (25, 26). Although the mechanism of corrosion control by sodium silicates is not well documented, the use of sodium silicates may be advantageous compared to phosphate-based treatments due to the lack of phosphorus (27). However, management requirements associated with LCR compliance have raised concerns with utilities regarding the use of sodium silicates for lead corrosion control (28).

Bacterial resistance to antibiotics and metals is often genetically linked, indicating mechanisms for co-selection of antibiotic resistance during exposure to metals such as copper or zinc (29). Several studies have reported opportunistic pathogens in drinking water systems that are chlorine resistant and contain ARGs encoding resistance to multiple types of antibiotics, which poses risks to human health and drinking water safety (30 – 32). Mobile genetic elements, such as class 1 integrons or broad-host-range plasmids, can readily jump from species or even phylum, thus increasing the probability of spreading ARGs and metal resistance genes (MRGs) to diverse bacteria or pathogens (33, 34). The molecular mechanisms for stimulating the horizontal transfer of ARGs by sub-inhibitory levels of metals, antibiotics, and disinfectants may involve reactive oxygen species (ROS) response systems and the SOS response pathway (12). Heavy metal ions, such as Cu(II), Ag(I), Cr(VI), and Zn(II), can induce oxidative stress and genotoxicity, and thus promote horizontal gene transfer of ARGs and co-selection of MRGs (12, 35). A wide spectrum of ARG and MRG pairs can be co-transferred via plasmids and integrons including bacitracin and Al/As/Cu/Cr/Fe/Hg/Te/Zn, aminoglycoside and Fe/Ni/Zn, tetracycline and Fe/Zn, and beta-lactam and As/Hg/Zn (36). The addition of corrosion inhibitors such as orthophosphates (especially zinc orthophosphate) and sodium silicates may also have impacts on antibiotic resistance and microbial communities in DWDS. There is little information available on the relative effects of zinc orthophosphate, sodium orthophosphate, and sodium silicates on the abundance of ARB, ARGs, and MRGs within drinking water systems. A previous study reported alterations in bacterial and fungal community structure in soils in response to the addition of 2 mM sodium silicate, in addition to decreased abundance of microbial taxa potentially containing pathogens (37). Therefore, the addition of sodium silicates to drinking water for corrosion control may also impact bacterial community composition, but has yet to be investigated.

The objective of this study was to quantify the impact of corrosion inhibitor type (zinc orthophosphate, sodium orthophosphate, and sodium silicates) on bacterial antibiotic and metal resistance abundance and microbial community structure in source water used for drinking water treatment. It was hypothesized that zinc orthophosphate dosing would increase the absolute and relative abundance of ARB and ARGs as compared to the sodium orthophosphate and sodium silicate treatments. This relationship was tested through laboratory-scale microcosms of a DWDS’s source water dosed with various corrosion inhibitors. ARB levels throughout the experiment were assessed by plating, and ARG concentration changes were quantified via quantitative polymerase chain reaction (qPCR). Microbial community diversity and composition changes in response to the corrosion inhibitor treatments were assessed using 16S rRNA gene amplicon sequencing of the V4 hypervariable region.

## MATERIALS AND METHODS

### Microcosm setup

Triplicate microcosm experiments were set up to test the impacts of three different corrosion inhibitors (zinc orthophosphate, sodium orthophosphate, and sodium silicate) on the abundance of ARB, ARGs, MRGs, and microbial community profiles in lake water that is used as a source water for multiple drinking water treatment plants. While microbial communities present in drinking water pipes are expected to be different from those present in the source water, microbial communities from pipes have already been exposed to corrosion inhibitors and disinfectants such as chlorine. It is also noted that the water chemistry of the source lake water is different from the water chemistry of drinking water after municipal treatment and in the distribution system. Moreover, every utility will have a different combination of influent microbial communities, drinking water treatment unit processes, and distribution system dynamics. These microbial communities collected from source water were selected to elucidate the potential, general, impacts of corrosion inhibitor types on antibiotic resistance.

Microcosm experiments with corrosion inhibitors were set up using 1 L glass bottles filled with 1 L of source water. Three different microcosm experiments (labeled Sets 1, 2, and 3) were conducted to determine the impact of corrosion inhibitor type and concentration on antibiotic resistance patterns in microbial communities. Within each set, the impacts of individual corrosion inhibitors were tested. Set 1 used source water collected from a local drinking water treatment plant (DWTP) water intake pipe (which brings in water from Lake Michigan approximately 1.25 miles from shore at a depth of 60 feet). Set 1 was amended with normal concentrations of corrosion inhibitors. It is referred to as “Deep” because the water was collected from the intake to a drinking water treatment plant that was deep in Lake Michigan. Set 2 used water collected off a breakwater structure at the beach at Atwater Park (Milwaukee, WI) approximately 50 meters into Lake Michigan and normal concentrations of corrosion inhibitors. Set 3 used water collected at the same location at Atwater Park and high concentrations of corrosion inhibitors. The source water for Sets 2 and 3 was labeled as “shallow” because they were collected from the surface of Lake Michigan. All samples were collected in a 45-L disinfected carboy for water homogeneity. The sample was transported immediately to the laboratory, where 1 L subsamples were aseptically added to sterile 1 L clear glass bottles.

Three corrosion inhibitors were used to dose the microcosms, and dosing occurred at two different concentrations to simulate different conditions in DWDS. The corrosion inhibitors included 99% purity zinc orthophosphate [Zn_3_(PO_4_)_2_] (Fisher Scientific, Waltham, MA), monobasic sodium orthophosphate (NaH_2_PO_4_) (98–102% purity; Fisher Scientific, Waltham, MA), and sodium metasilicate (Na_2_SiO_3_) (49.5–52.5% Na_2_O; Sigma Aldrich, St. Louis, MO). Sodium metasilicate was used as a representative silicate-based corrosion inhibitor. Previous studies have suggested that different sodium silicate formulations may have different efficacy for lead corrosion control (28). A national survey of U.S. drinking water utilities reported concentrations of phosphate corrosion inhibitors ranging from 0.2 to 3.0 mg/L in DWDS, with over 50% of utilities dosing between 0.7 and 2.0 mg/L as PO_4_ (17). Microcosm Sets 1 and 2 were given corrosion inhibitors at concentrations to simulate full-scale DWDS conditions, as follows: 1 mg/L zinc orthophosphate as PO_4_, 1 mg/L sodium orthophosphate as PO_4_, and 10 mg/L sodium silicate as SiO_2_. Typical recommended dosages of sodium silicates for water treatment purposes range from 8 to 55 mg/L as SiO_2_ (22, 24). The concentrations used in these sets were labeled as “normal.” In Microcosm Set 3, high levels of corrosion inhibitors were added as follows: 10 mg/L zinc orthophosphate as PO_4_, 10 mg/L sodium orthophosphate as PO_4_, and 100 mg/L sodium silicate as SiO_2_. The concentrations used in Set 3 were labeled as “high.” All microcosm treatment groups were performed in triplicate and operated for a duration of 7 days. It is noted that the impacts of corrosion inhibitors in DWDS are most relevant in *in situ* pipe biofilms, where organisms are exposed to inhibitors for long term. A limitation to these lab experiments is that variables were controlled to isolate the impact of corrosion inhibitor. Long-term *in situ* pipe studies could provide information on what happens at full scale, albeit with less control. All microcosms were placed on an orbital mixer at 150 rpm to homogenize the water and keep the cells suspended. The microcosms were incubated at room temperature (25.0 ± 2.0°C) in the dark. Water samples were collected on days 0, 3, and 7 for analysis of microbiological and chemical constituents.

### Quantification of antibiotic-resistant heterotrophic bacteria

Water was collected from each of the microcosms on days 0, 3, and 7 and plated on R2A media with and without antibiotics to determine the abundance of antibiotic resistant heterotrophic bacteria. R2A media plates were amended with 100 µg/mL of cycloheximide to inhibit fungal growth. Antibiotics were added to R2A at concentrations based on the Clinical and Laboratory Standards Institute guidelines (38). The antibiotics tested were ampicillin (AMP; 32  µg/mL), ciprofloxacin (CIP; 4  µg/mL), rifampicin (RIF; 4  µg/mL), sulfamethoxazole (SULF; 100  µg/mL), tetracycline (TET; 16 µg/mL), trimethoprim (TRIM; 16 µg/mL), and vancomycin (VAN; 32 µg/mL). The R2A plating approach yields growth of mixed communities. It is not possible to distinguish individual strains that acquired resistance from shifts in microbial communities that contain bacteria with more intrinsic resistance. Results are used to quantify if more or less resistant bacteria were present after exposure without determining mechanisms. All plates were done in triplicate and incubated for 5 days at 30°C, according to the protocol for plate growth in an incubator with a container of tap water to maintain the relative humidity and prevent plate dehydration (21, 39). After 5 days, the colony-forming units on each plate were counted manually.

### Quantification of ARGs, MRGs, and class 1 integrons

Water from each microcosm was measured in a graduated cylinder and harvested by vacuum filtration onto a 0.22-µm Millipore Express PLUS Membrane filter (MilliporeSigma, Burlington, MA). Filters were placed in sterile 2 mL tubes and stored at −20°C until DNA was extracted. FastDNA Spin Kits (MP Biomedicals, Solon, OH) were used for DNA extraction as described previously (10). DNA yield was determined by microspectrophotometry with a Nanodrop One (Thermo Scientific, Waltham, MA). Genes in the extracted DNA were enumerated by qPCR using primers previously published for bacterial 16S rRNA (40), the ARGs *bla*_TEM_ (41), *sul*1 (42), *sul*2 (21), and *qac*EΔ1 (43), the MRGs *cop*A (44), *czc*C (44), *czc*D (44), and the integron *intI*1 (45). Results were reported as copies per liter and normalized to 16S rRNA gene copies to observe the trends in relative abundance. The qPCR limit of quantification was determined for each gene according to MIQE guidelines (46). An additional description of qPCR methodology is included in the Supplementary Information and specific primer sets, annealing temperatures, efficiencies, and detection limits are described in Table S1.

### PCR, amplicon sequencing, and sequence processing

Microbial communities from water samples were prepared for analysis by triplicate PCR amplifying and pooling V4 hypervariable regions of 16S rRNA genes (10, 47). V4 primers were 515F (GTGYCAGCMGCCGCGGTAA) (48) and 806R (GGACTACNVGGGTWTCTAAT) (49). PCR amplicons were sequenced with Illumina MiSeq 2 × 250 paired-end chemistry at the Great Lakes Genomics Center (http://greatlakesgenomics.uwm.edu). Primer and barcode sequences were removed from reads using cutadapt (50). Reads were processed using the R (51) package DADA2 (52). Specifically, 16S rRNA gene reads were filtered (maxEE = 2, maxN = 0, and truncQ = 10), merged, error corrected, and chimera checked. Merged pairs with sequence lengths 5% above (266 bp) and below (240 bp) the median sequence length (253) were kept. Taxonomy was assigned to the amplicon sequence variants (ASVs) using DADA2 from the SILVA v. 138 reference database (53).

### Water quality analysis

Water quality for the source drinking water was characterized on the day of collection (day 0) for the following parameters: alkalinity, pH, hardness, dissolved oxygen, ammonia, phosphate, dissolved metals, silicate, and dissolved organic carbon (DOC). Water was aseptically removed from the microcosms on days 3 and 7, and analyzed for pH, dissolved metals, DOC, phosphate, and silicate. A Thermo Fisher Scientific pH probe was used for measuring pH for the microcosms. Sodium silicate, alkalinity, hardness, dissolved oxygen, and ammonia were measured according to appropriate standard methods using Hach kits (Hach Company, Loveland, CO). Phosphate was measured using the ascorbic acid method (Standard Method 4500-P) (54). Dissolved metal concentrations were quantified using an Agilent 7700s inductively coupled plasma mass spectrometer (Agilent Technologies, Santa Clara, CA). Water samples analyzed for DOC were filtered with 0.45 µm polytetrafluoroethylene (PTFE) filters prior to acidification to <2 pH with HCl (36.5–38.0%) and quantification using a Shimadzu TOC analyzer (Shimadzu, Kyoto, Japan). Water quality measurements are included in Tables S2 and S3.

### Statistical analysis

Statistical analysis was performed using GraphPad Prism V 9.3.1 (α < 0.05). A two-way Analysis of variance (ANOVA) was used to determine significant differences among the concentrations and treatment types for absolute and relative abundance measurements of ARB, ARGs, MRGs, and *intI*1 on days 0, 3, and 7. *Post hoc* multiple pairwise comparisons were conducted using Tukey’s honest significant differences test.

Differences between microcosm microbial communities were measured in a pairwise Bray-Curtis distance matrix generated by the *vegdist* function in R (51) package *vegan* (55). To test the significance that treatments affected microbial communities, a permutational multivariate analysis of variance (PERMANOVA, also in *vegan*) was performed on the distance matrix, constricting permutations within source water strata. Assessing the relative significance of treatments was done with a *post hoc* pairwise multilevel comparison on the PERMANOVA with the R package *pairwiseAdonis* (56). The extent to which a treatment changed a community was measured by comparing Bray-Curtis distances between treatment samples and respective same-day controls. Triplicate samples were statistically similar (PERMANOVA, *P* = 1.0; Table S4) and distances among them were low (0.21 ± 0.058; Table S5); therefore, distances among controls were assumed to represent the “expected change” communities underwent in the absence of inhibitors. To compare the extent of change between treatments, first nonparametric distributions of Bray-Curtis values were confirmed with the R function *shapiro.test*; then, *P* values from Wilcoxon-Mann-Whitney tests were obtained with the R function *wilcox.test*. Correlations between ASV and ARG abundances were explored using the Spearman method in the R function *cor.test*.

ASVs were subsetted to include only those that were present in each of the triplicate microcosms. Among those, any classified as genera associated with waterborne pathogens (*Mycobacterium* [57]*, Legionella* [58]*, Arcobacter* [59]*,* and *Pseudomonas* [60]) and pipe corrosion (*Leptothrix* [61]*, Pseudomonas* [62, 63]*, Shewanella* [64, 65]*, Sulfuricurvum* [66]*, Acidithiobacillus* [67]*, Geothermobacter* [68]*, Thiobacillus* [69]*, Geobacter* [70]*, Desulfomicrobium* [71]*, Desulfobulbus* [72]*, Sulfurospirillum* [73]*,* and *Desulfosporosinus* [74]) were analyzed. Treatment response was estimated as the log2 fold change between the mean relative abundances of genera in treatment microcosms to controls on day 7 (Tables S6 and S7). Data and code for the DNA sequence analysis can be found on GitHub (https://github.com/loulanomics/Microcosm_corrosionInhibitors).

## RESULTS AND DISCUSSION

### Impacts on heterotrophic antibiotic-resistant bacteria

#### Impact of corrosion inhibitor type and concentration

**Fig 1:**
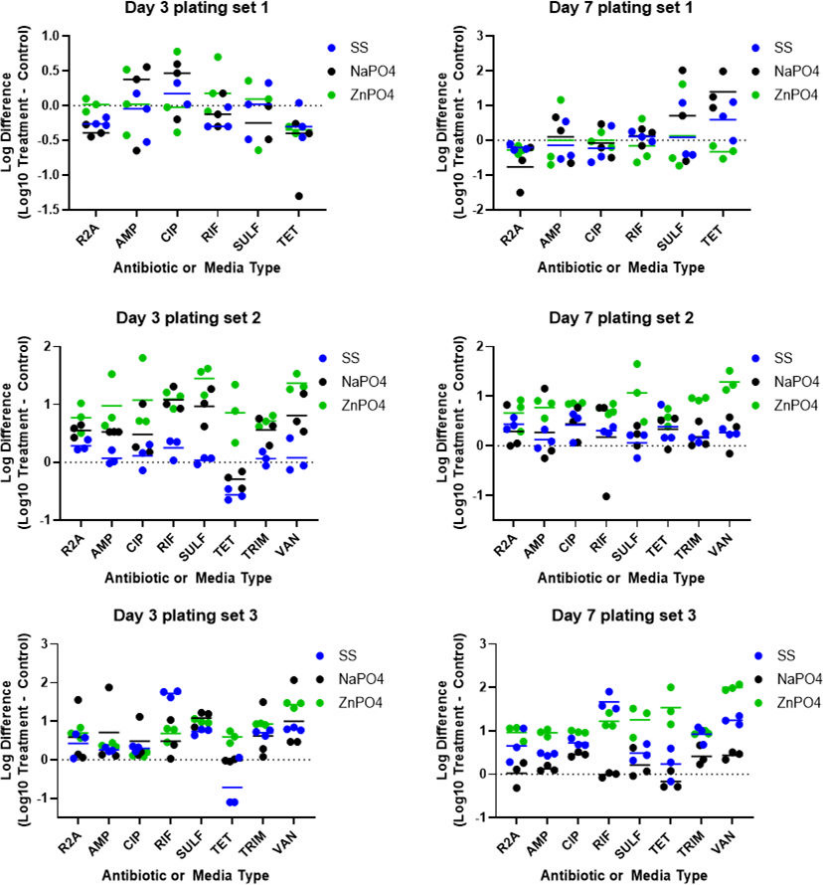
Log difference in absolute abundance of total heterotrophic bacteria (R2A) and antibiotic resistant bacteria on day 3 and day 7 based on direct plating from microcosms containing different types and concentrations of corrosion inhibitors. The difference in colony-forming unit (CFU), counts as log_10_ per liter of water for each treatment microcosm, was determined relative to the control microcosms on days 3 and 7. The type of media or antibiotic is denoted on the x-axis. Corrosion inhibitor type is indicated by color. No difference from control is indicated by the dashed line. Triplicate microcosm values are indicated by points and the average value for each triplicate set is indicated by a solid line. Microcosm Sets 1 and 2 were dosed with a normal concentration (1×) of corrosion inhibitors and Set 3 was dosed with high concentrations (10×) of corrosion inhibitors. SS, sodium silicate; NaPO_4_, sodium phosphate; ZnPO_4_, zinc orthophosphate.

ARB were quantified in water samples collected from each of the microcosms on days 3 and 7 to determine the effect of corrosion inhibitors on the abundance of phenotypic antibiotic resistance in the microbial communities. A limitation to this method is that the underlying mechanism for changes has not been elucidated. For example, it cannot be determined if a specific microbe acquired resistance and then grew or if there was selection for bacteria with intrinsic resistance. There was a significantly greater absolute abundance of ARB in the zinc orthophosphate treatment at 1 mg/L compared to untreated controls on day 3 for ARB resistant to AMP, CIP, RIF, SULF, TET, TRIM, and VAN (two-way ANOVA; all *P* values < 0.05) (Fig. 1, Set 2). Average CIP resistant bacteria increased by 12-fold (over 1,000%), while average VAN-resistant bacteria also increased by 12-fold. Sodium orthophosphate treatment at 1 mg/L also resulted in significantly greater absolute abundance of RIF, SULF, TRIM, and VAN compared to untreated controls by day 3 (two-way ANOVA; all *P* values < 0.05) (Set 2). Both zinc orthophosphate and sodium orthophosphate treatments at 1 mg/L resulted in significantly increased absolute abundance of total heterotrophic bacteria (R2A) (two-way ANOVA; *P* values < 0.05). At high concentrations (10 mg/L), the sodium orthophosphate treatment also resulted in significantly greater absolute abundance of ARB resistant to SULF compared to untreated controls (two-way ANOVA; *P* value < 0.05) (Set 3). Similarly, there was a significantly greater absolute abundance of ARB in the zinc orthophosphate treatment at 10 mg/L compared to untreated controls on day 3 for bacteria resistant to SULF and VAN (two-way ANOVA; *P* values < 0.05). The average increase in VAN resistant bacteria was 26-fold. Sodium silicate (100 mg/L) treated communities demonstrated significantly greater resistance to RIF and SULF by day 3. At normal concentrations found in DWDS (10 mg/L as SiO_2_), sodium silicate treatment did not select for increased absolute or relative abundance of ARB relative to untreated controls by day 3. However, high levels of sodium silicate (100 mg/L as SiO_2_) resulted in significantly increased abundance of ARB resistant to RIF, TRIM, and VAN (two-way ANOVA; *P* values < 0.05). The increase in VAN resistant bacteria was sixfold.

By day 7, the absolute abundance of ARB was significantly greater in the 1 mg/L zinc orthophosphate treatment for CIP, SULF, TRIM, and VAN as compared to the untreated controls (two-way ANOVA; all *P* values < 0.05) (Fig. 1, Set 2). The average increases were nearly one order of magnitude (10-fold, as indicated by a value of 1 on the y-axis). The zinc orthophosphate treatment at 1 mg/L also resulted in significantly greater ARB resistant to TRIM and VAN compared to sodium orthophosphate treatment at 1 mg/L and sodium silicate treatment at 10 mg/L (two-way ANOVA; all *P* values < 0.05) (Set 2). The addition of 10 mg/L zinc orthophosphate on day 7 resulted in the highest ARB growth of all the tested conditions (Set 3), with significantly higher absolute abundances of total heterotrophic bacteria (R2A) and ARB resistant to AMP, CIP, RIF, SULF, TET, TRIM, and VAN when compared to the untreated controls (two-way ANOVA; all *P* values < 0.05). Sodium orthophosphate addition at 10 mg/L (Set 3) on day 7 exhibited a significantly greater absolute abundance of ARB resistant to CIP and VAN as compared to the untreated controls (two-way ANOVA; *P* values < 0.05). In contrast, the sodium silicate treatment at 10 mg/L (Sets 1 and 2) did not result in any selection for ARB by day 7. However, the absolute abundance of ARB was significantly greater for the sodium silicate treatment at 100 mg/L (Set 3) by day 7 for AMP, CIP, RIF, and VAN compared to the untreated controls (two-way ANOVA; *P* values < 0.05).

Analyzing the change in relative ARB abundance (ARB CFUs/total bacteria CFUs) provides a way to distinguish increased bacterial growth overall from increased ARB as a proportion of the microbial community. When ARB counts were corrected for total heterotrophic bacteria, a significantly greater relative abundance of VAN-resistant bacteria was observed in the 10 mg/L zinc orthophosphate treatment compared to the untreated control by day 3 (two-way ANOVA; *P* value < 0.05) (Fig. S1 in the Appendix). Increased VAN resistance was also observed at 1 mg/L zinc orthophosphate, but the differences were not statistically significant due to larger inter-microcosm variability. There was also a significant increase in the relative abundance of VAN-resistant bacteria in the zinc orthophosphate compared to the sodium silicate treatment at 1 mg/L (two-way ANOVA; *P* value < 0.05). Sodium silicate addition at higher dosages (100 mg/L) significantly increased the relative abundance of RIF-resistant bacteria compared to untreated controls and both phosphate corrosion inhibitor treatments by day 3 (two-way ANOVA; *P* values < 0.05). By day 7, the changes in relative abundance for each of the corrosion inhibitor treatments were not significantly different from the untreated controls (Fig. S2 in the Appendix).

The increase in abundance of total heterotrophic bacteria and ARB indicates that the addition of zinc orthophosphate and sodium orthophosphate may be providing nutrients that were limiting to the bacterial communities. Zinc is an essential trace metal and can stimulate microbial growth in aquatic systems (21). Bacterial exposure to zinc at increased concentrations may stimulate metal resistance mechanisms and co-select for antibiotic resistance within bacterial populations (75). Orthophosphate is readily used by heterotrophic bacteria, and previous studies have documented increased microbial growth after phosphate additions ranging from 0.03 to 3 mg/L (19, 76). Similarly, zinc orthophosphate addition at 1–3 mg/L as PO_4_ increased microbial community diversity and richness in drinking water biofilms grown on lead and copper coupons (20). The results observed in this study indicate that the addition of zinc orthophosphate and sodium orthophosphate resulted in increased positive selection for heterotrophic bacteria resistant to multiple types of antibiotics at concentrations typically used for corrosion control in DWDS (1 mg/L as PO_4_). Overall, the high-dosed microcosms (Set 3) exhibited the highest absolute abundances of ARB. Sodium silicate addition at the high dose (100 mg/L) also resulted in increased selection for ARB resistant to multiple types of antibiotics. These results suggest that corrosion inhibitors may play an important role in bacterial communities and the prevailing levels of antibiotic resistance in DWDS.

Out of all the corrosion inhibitors tested, zinc orthophosphate treatment resulted in the highest observed growth of ARB. The differences in ARB growth between zinc orthophosphate and sodium orthophosphate indicate zinc is a growth promoter for ARB resistant to multiple classes of antibiotics. The high-level dosage of zinc orthophosphate resulted in the highest observed growth of ARB resistant to multiple types of antibiotics. The increase in ARB relative to untreated controls was greater for each of the corrosion inhibitor types at high doses (10 mg/L as PO_4_ and 100 mg/L as SiO_2_) compared to the normal doses (1 mg/L as PO_4_ and 10 mg/L as SiO_2_). At high levels, the corrosion inhibitors serve as a nutrient source for microorganisms and ARB resistant to multiple types of antibiotics. The addition of silicates consistently resulted in lower levels of ARB compared to zinc and sodium orthophosphate addition.

The growth of ARB was greater by day 7 compared to day 3 for all of the conditions tested. The increased time of exposure of bacterial populations to the different corrosion inhibitor treatments may have caused the resistant populations that developed by day 3 through intrinsic and/or acquired resistance mechanisms to be outcompeted by other bacteria by day 7. Similarly, a previous study suggested that the selection of ARB was transient in nature due to the initial influx of zinc and assimilation through population growth (21). Interestingly, the addition of orthophosphates at normal concentrations (1 mg/L) resulted in larger increases in the absolute abundance of ARB on day 3 as compared to the higher 10 mg/L treatment. However, the transient selection by sodium orthophosphate addition for ARB observed on day 3 did not exhibit the same patterns of selection of ARB by day 7.

### Impact on genotypic antibiotic resistance

#### Impact of corrosion inhibitor type and concentration

**Fig 2:**
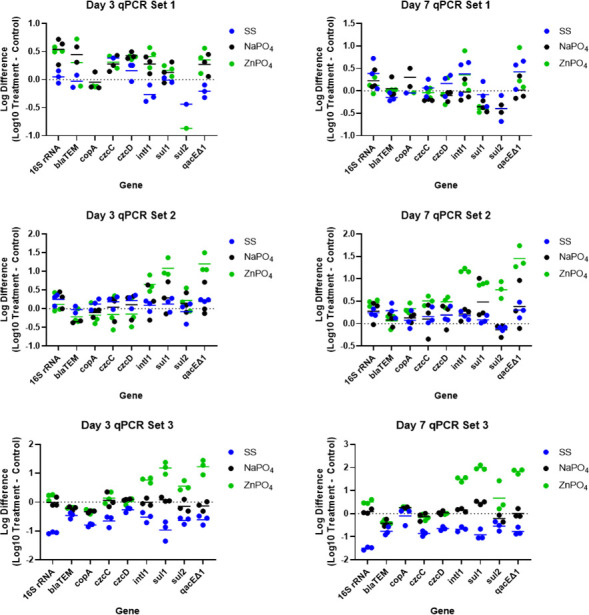
Log difference in absolute abundance of total bacterial biomass (16S rRNA), ARGs, MRGs, and i*ntI*1 on days 3 and 7 based on qPCR analysis from microcosms containing different types and concentrations of corrosion inhibitors. The difference in counts of gene copies in Log_10_ per liter of water for each treatment microcosm was determined relative to the control microcosm on days 3 and 7, which is shown on the y-axis. The type of gene is denoted on the x-axis. Corrosion inhibitor type is indicated by color. Different experimental conditions included the following: Set 1, normal concentration (1×) of corrosion inhibitors, lake water from DWTP intake pipe; Set 2, normal concentration (1×) of corrosion inhibitors, lake water collected at recreational beach; Set 3, high concentration (10×) of corrosion inhibitors, lake water collected at recreational beach.

ARGs, MRGs, and the integrase gene of class 1 integrons (*intI*1) were quantified in water samples collected from each of the microcosms on days 3 and 7 to determine the effect of corrosion inhibitors on the abundance of genotypic antibiotic resistance and metal resistance. There was a significantly greater absolute concentration of *sul*1 and *qac*EΔ1 in the zinc orthophosphate treatment at 1 mg/L compared to all other treatments on day 3 (two-way ANOVA; *P* values < 0.05) (Fig. 2; Set 2). Even more ARGs had higher concentrations than the control in the 10 mg/L zinc orthophosphate addition; these genes included *cop*A, *intI*1, *sul*1, *sul*2, and *qac*EΔ1 (two-way ANOVA; all *P* values < 0.05). Sodium orthophosphate addition at 1 mg/L did not result in significant increases in ARG concentrations by day 3, but the 10 mg/L addition increased the absolute abundance of *bla*_TEM_ and *cop*A to levels significantly higher than untreated controls (two-way ANOVA; *P* values < 0.05). Sodium silicate addition at 10 mg/L did not result in any significant changes in ARG or MRG abundance relative to untreated controls by day 3. Sodium silicate addition at high concentrations (100 mg/L) selected for significantly lower absolute abundance of 16S rRNA genes and ARGs including *bla*_TEM_, *cop*A, *czc*C, *sul*1, *sul*2, and *intI*1 by day 3 compared to untreated controls, sodium orthophosphate, and zinc orthophosphate treatments (two-way ANOVA; *P* values < 0.05).

By day 7, zinc orthophosphate treatment at 1 mg/L resulted in significantly greater absolute abundance of *sul*1, *sul*2, *qac*EΔ1, and *intI*1 compared to untreated controls (two-way ANOVA; all *P* values < 0.05) (Fig. 2; Set 2). Zinc orthophosphate treatment at 10 mg/L also resulted in a significantly greater absolute abundance of 16S rRNA genes and *sul*1, *qac*EΔ1, and *intI*1 as compared to untreated controls (two-way ANOVA; *P* value < 0.05). Sodium orthophosphate addition did not exhibit similar growth patterns compared to zinc orthophosphate at the same concentrations tested, indicating that the addition of zinc may be promoting an increased abundance of antibiotic resistance within the microbial communities. Zinc is an essential micronutrient required for proper structure and function of many proteins in bacterial cells (77). The addition of zinc may also promote P scavenging in bacterial communities since bacterial cells can require zinc for production of phosphatase enzymes (e.g., alkaline phosphatase) (78). A previous study observed similar operons in several different bacterial species containing genes for heavy metal translocating ATPases and phosphatases (79). Communities without zinc orthophosphate addition may have been lacking sufficient zinc for phosphatase formation, thus limiting P scavenging and bacterial growth. Similarly, Kappell et al. (80). observed similar selection for ARB and ARGs with both zinc chloride and zinc orthophosphate addition, indicating zinc as the selecting agent for observed increased resistance in the microbial communities. Sodium silicate addition at 100 mg/L significantly decreased the absolute abundance of several genes including 16S rRNA and *czc*C, *czc*D, *sul*1, *sul*2, and *intI*1 compared to untreated controls, sodium orthophosphate, and zinc orthophosphate treatments (two-way ANOVA; *P* values < 0.05), which was similar to results observed on day 3.

Analyzing the change in relative abundance of resistance genes provided an indication of genotypic selection in the presence of different corrosion inhibitor types and concentrations. When normalized to 16S rRNA gene copies, there was a significantly greater relative abundance of *sul*1, *qac*EΔ1, and *intI*1 gene copies in the zinc orthophosphate treatment at 1 mg/L compared to untreated controls, sodium silicate, and sodium orthophosphate by day 3 (two-way ANOVA; *P* values < 0.05) (Figure S3). Similar selection for ARGs was observed at 10 mg/L zinc orthophosphate, which also exhibited significantly greater relative abundance of *sul*1, *qac*EΔ1, and *intI*1 compared to untreated controls and sodium orthophosphate by day 3 (two-way ANOVA; *P* values < 0.05). Sodium orthophosphate addition at 1 and 10 mg/L did not result in any significant changes in relative abundance of ARGs, MRGs, or *intI*1 relative to untreated controls by day 3. Sodium silicate addition at higher dosages (100 mg/L) significantly increased the relative abundance of *intI*1 compared to untreated controls and sodium orthophosphate by day 3 (two-way ANOVA; *P* values < 0.05).

By day 7, there was a significantly greater relative abundance of *intI*1 and *qac*EΔ1 in the zinc orthophosphate compared to untreated controls and sodium orthophosphate addition (Figure S8) at 1 mg/L (two-way ANOVA; *P* values < 0.05). Similar selection was observed at 10 mg/L zinc orthophosphate, which exhibited significantly greater relative abundance of *intI*1, *sul*1, and *qac*EΔ1 by day 7 (two-way ANOVA; *P* values < 0.05). Similar to the results from day 3, sodium orthophosphate addition at 1 and 10 mg/L did not result in any significant changes in relative abundance of ARGs, MRGs, or *intI*1 relative to untreated controls. Interestingly, by day 7 sodium silicate addition at 100 mg/L resulted in a significantly increased relative abundance of several genes including *bla*_TEM_, *czc*D, *cop*A, *intI*1, and *sul*2 compared to untreated controls and sodium orthophosphate addition (two-way ANOVA; *P* values < 0.05).

Zinc orthophosphate addition at concentrations ranging from 1 to 10 mg/L resulted in the highest observed growth of ARB and ARGs. The sub-inhibitory concentrations of zinc allowed a growth or survival advantage for bacteria harboring the *sul*1, *sul*2, *qac*EΔ1, and *intI*1 genes. Similar to the changes observed in abundance of antibiotic resistant heterotrophic bacteria, the presence of zinc and not orthophosphate was determined to be the major factor contributing to the selection of ARGs and *intI*1. The integrase gene of class 1 integrons (*intI*1) plays an important role in the proliferation of acquired resistance to antibiotics, metals, and disinfectants through the ability to incorporate gene cassettes (81, 82). The *sul*1 and sul2 genes encode dihydropteroate synthase that are not inhibited by sulfonamides (83). The *sul*1 gene is normally linked to other resistance genes in class 1 integrons, while *sul*2 is usually located on small nonconjugative plasmids or large transmissible multi-resistance plasmids (83, 84). The *qac*EΔ1 gene encodes resistance to quaternary ammonium compound disinfectants and is commonly observed on the same mobile resistance gene cassettes as *sul*1 and *intI*1 (85).

The metal resistance genes targeted in this study, *czc*C, *czc*D, and *cop*A, were targeted because they indicate the presence of zinc and other related metal-resistance mechanisms (86). The lack of significant changes in gene abundance for zinc-related resistance genes may support the evidence that low environmental zinc concentrations are limiting microbial growth for communities that are not treated with zinc orthophosphate. The addition of zinc led to positive selection for bacteria to be able to influx zinc, rather than zinc negatively selecting against bacteria without metal resistance (21). Zinc is an essential nutrient for bacterial cells and plays an important role in many cellular functions and metabolic pathways including phosphatase regulation (78). Protein phosphatases are involved in metabolic homeostasis, stress response, and other essential biological mechanisms. In fact, Zn(II) is required for initial phosphate binding and for the formation of the phosphoseryl intermediate (78). In the current study, zinc orthophosphate treated communities were likely more efficient at scavenging P, which increased bacterial growth compared to sodium orthophosphate or sodium silicate treated communities. Additionally, it is hypothesized that the observed growth was by bacteria with intrinsic resistance, or selection by bacteria that either expressed different resistance genes for zinc than were targeted in the study, or were not affected negatively by the addition of zinc.

Previous studies have investigated the impacts of zinc with respect to impacts on microbial community composition in a variety of different environments. High levels of metals, including zinc, can be toxic to bacteria (87). However, several studies have demonstrated that subinhibitory concentrations of zinc can promote increased antibiotic resistance and bacterial growth. For example, a previous study investigated and revealed that heavy metals including Cu(II), Ag(I), Cr(VI), and Zn(II), at environmentally relevant and subinhibitory concentrations, promoted conjugative transfer of ARGs between *E. coli* strains (12). The mechanisms of this phenomenon were attributed to increases in intracellular ROS formation, SOS response, increased cell membrane permeability, and altered expression of conjugation-relevant genes. Similarly, another study observed increased growth of ARB resistant to ciprofloxacin, streptomycin, trimethoprim, and erythromycin, in addition to increased levels of ARGs such as *sul*1, *sul*2, *qac*H, and *intI*1 in response to zinc exposure (21). Additionally, zinc ions and zinc nanoparticles at low concentrations can promote substantial ROS production and increased *amo*A gene expression in nitrifying bacteria (88). The results of this study also support the hypothesis that zinc orthophosphate exposure can result in increased growth of ARB and ARGs in bacterial communities. Consequently, it is imperative to gain a better understanding of the impacts of corrosion inhibitors such as zinc orthophosphate on microbial ecology and antibiotic resistance proliferation in distribution systems in laboratory, pilot-scale, and full-scale settings.

A limitation to the findings presented here is that, as explained in the Materials and Methods, the experimental setup had different conditions than those found in real world distribution systems. Source water was used to not bias the microbial community through selection in treatment plant unit operations. Therefore, the microbial communities used in these experiments were different than those present in distribution systems, similar to how the water chemistry is different. Each drinking water utility has a unique combination of source water, treatment process, and distribution system dynamics. These findings provide an indication that corrosion inhibitors behave differently but should not be used to infer precisely what will happen or is happening in a full-scale system that has several dynamics occurring at once that impact antibiotic resistance. These results indicate that corrosion inhibitor type matters, and future research experiments should include microcosms with metals and corrosion products that can interact with the corrosion inhibitors to determine effects on antibiotic resistance.

### Impact of source water on antibiotic- and metal-resistance profiles

The impact of source water selection was evaluated by comparing results from microcosm experiment Sets 1 and 2, which were both operated under typical corrosion inhibitor concentrations found in full-scale DWDS (i.e., 1 mg/L as PO_4_ and 10 mg/L as SiO_2_ dosages) but had different source waters. In Set 1, the source water collected from the DWTP influent (i.e., deep source water) displayed different phenotypic resistance patterns than the surface water collected for Set 2 from Atwater Beach (i.e., shallow source water) (Fig. 1). There were no observed significant changes in absolute or relative abundance for ARB for the samples collected from the DWTP in experiment Set 1 relative to untreated controls. However, there were significant changes in abundance observed for several ARGs in Set 1, especially for zinc orthophosphate and sodium orthophosphate treated communities. The DWTP intake pipe extends more than 2 miles from the shore and collects lake water at a depth of approximately 60 feet. The bacterial community and concentrations of contaminants present in this water sample were lower than those in the source water collected for Microcosm Set 2. The source water from Atwater Beach is likely more representative of the surrounding landscape with increased amounts of nutrients, bacteria, and other contaminants from stormwater runoff entering the lake and continuous mixing with the sand and sediment at the beach shore.

The source water collected for microcosm experiment Set 2 contained >0.05 mg/L of orthophosphate on day 0, but the day 0 sample collected from Microcosm Set 1 was below the detection limit for orthophosphate (<0.05 mg/L as PO_4_). The silicate concentration for Set 1 was below the detection limit (<1 mg/L as SiO_2_); however, the silicate concentration for Set 2 on day 0 was approximately 2 mg/L as SiO_2_. The source water for Set 2 also had slightly higher DOC values ranging from 2.05 to 2.99 mg/L compared to 2.16 to 2.92 mg/L for Set 1. DOC in water bodies sourced for drinking water range from 0.1 to 20 mg/L (89). Source water and initial community composition clearly can make a difference, as this is a response to the addition of a simple compound. Additionally, these are short term responses; it will be of interest for long-term selection if corrosion inhibitors are added at low doses for long periods of time. The observed patterns in antibiotic- and metal-resistance profiles indicate community composition with nutrient dynamics playing a role in dictating outcomes.

### Impact of corrosion inhibitors on microbial community structure

**Fig 3:**
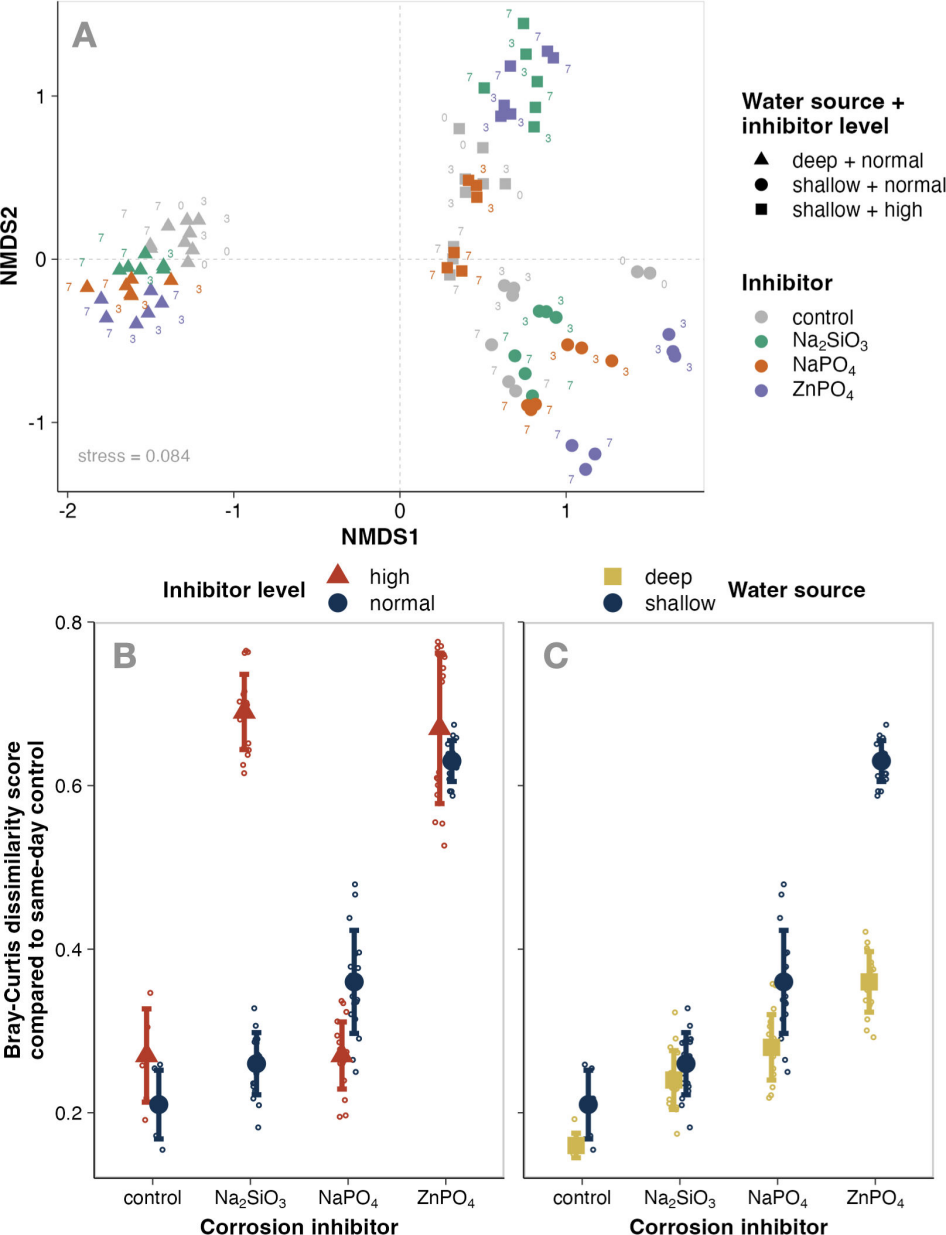
Extent of microbial community change induced by corrosion inhibitors in microcosm experiments. Panel A visualizes Bray-Curtis distances between samples in an ordination using nonmetric multidimensional scaling (NMDS), with point shapes indicating the experiment (triangles = deep source water with normal inhibitor levels, circles = shallow source water with normal inhibitor levels, and squares = shallow source water with high inhibitor levels), point colors indicating the corrosion inhibitor appended to experiments (grey = control, green = sodium silicate, orange = sodium phosphate, and purple = zinc phosphate), and numbers next to points showing the incubation day that sample was collected. In panels B and C, the x-axis shows experiments wherein microbial communities from deep (yellow squares) or shallow (blue circles) water were incubated in microcosms appended with corrosion inhibitors such as sodium silicate (Na₂SiO₃), sodium phosphate (NaPO_4_), or zinc orthophosphate (ZnPO_4_). Points represent a comparison between an experiment and its respective control (source community without appendments) at the end of incubations (day 7). Point heights on the y-axis dictate the Bray-Curtis dissimilarity score between the experiment and the control. Panel A shows the extent of change induced by corrosion inhibitors on shallow source water communities at normal corrosion inhibitor concentrations. Panel B shows the extent of change with corrosion inhibitor concentrations one order of magnitude greater than normal (“high”).

Both the type of inhibitor and the level at which it was appended to microcosms significantly altered microbial community compositions (PERMANOVA, *P* < 0.001; Table S4). Expectedly, when source water was collected on day 0, deep water communities were distinct from shallow, separating mostly along the NMDS1 axis in ordination (Fig. 3A). As treatment progressed, communities in the two shallow water experiments became more dissimilar, with high inhibitor levels dispersing them in the positive direction along NMDS2 and normal inhibitor levels in the negative direction.

Overall, zinc orthophosphate had the greatest impact on community profiles (pairwise multilevel comparison, *P* = 0.001), followed by sodium silicate (*P* = 0.005) and sodium phosphate (*P* = 0.049). Communities were more impacted by high inhibitor levels than normal inhibitor levels, but these trends were inhibitor-specific. At normal inhibitor levels, zinc orthophosphate induced the most compositional changes (Wilcoxon-Mann-Whitney, *P* = 2.7^e-10^) and sodium silicate the least (*P* = 4.7^e-05^; Fig. 3B). In contrast, at high levels, both sodium silicate and zinc orthophosphate were equally impactful (Wilcoxon-Mann-Whitney, both *P* = 7.4^e-6^), while sodium orthophosphate-induced change was negligible (*P* = 3.9).

Deep-water microbial communities demonstrated a greater resistance to inhibitor-induced change (Fig. 3C). In experiments with zinc phosphate, distances from controls in deep water communities were nearly half of those in shallow water. The impact that sodium phosphate had on shallow water communities was also significantly greater than deep (Wilcoxon-Mann-Whitney, *P* = 2.2^e-10^), but the effects of sodium silicate were similar between water sources (*P* = 0.091). Deep water microbial communities’ resistance to change was further evident in ordination (Fig. 3A). At normal inhibitor levels, the dispersion of shallow water communities (NMDS1 = 1.1, NMDS2 = 1.2) was nearly double the dispersion of deep-water communities (NMDS1 = 0.67, NMDS2 = 0.64).

### Fate of bacteria associated with corrosion and pathogens

**Fig 4:**
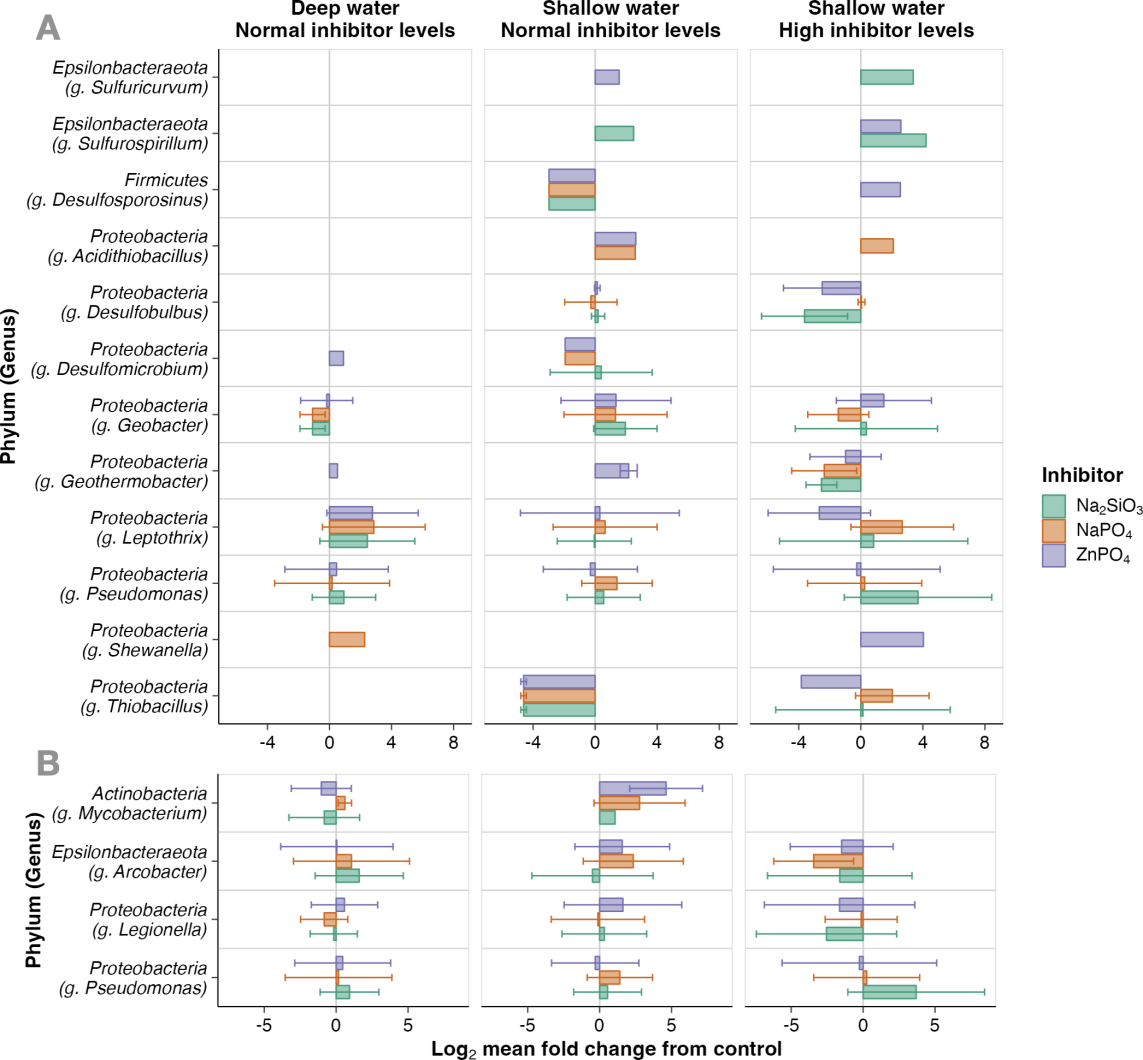
The response of bacteria associated with (**A**) pipe corrosion and (**B**) pathogens to corrosion inhibitors. Response was estimated as the log2 fold change (x-axis) of ASV (y-axis) relative abundances between treatment microcosms and controls. Columns extend to the mean log fold change of ASVs in that genus; error bars extend to the minimum and maximum standard deviation from the mean. Treatment microcosms were (left) Set 1, deep source water microbial communities appended with corrosion inhibitors at normal concentrations; (middle) Set 2, shallow source water communities appended with normal levels of corrosion inhibitors; or (right) Set 3, shallow source water communities appended with corrosion inhibitors at levels one magnitude greater than normal (“high”). Amendments were either sodium silicate (green, Na₂SiO₃), sodium phosphate (orange, NaPO_4_), or zinc phosphate (purple, ZnPO_4_).

ASVs were selected based on their classifications as genera associated with pipe corrosion (Table S6; Fig. 4). Only ASVs that were present in each triplicate microcosms were analyzed (2,728 out of 10,310). In microcosms from deep source water (Set 1), 535 ASVs out of 1,378 were classified to the genus level, and 10 were associated with pipe corrosion. Shallow source water communities with normal inhibitor levels (Set 2) had 718 out of 1,447 ASVs classified to genus level, and 20 associated with pipe corrosion. Shallow source water with high levels of corrosion inhibitors (Set 3) had 24 ASVs associated with corrosion, out of the 759 ASVs that were classified to the genus level. Their response to treatment was inferred by the log2 fold change between the mean relative abundances in control and treatment microcosms.

Most genera had indicator ASV abundances increase or decrease throughout the treatment to varying degrees (Table S6; Fig. 4A). Consistently enriched genera included *Shewanella* (log2 fold change = 3.2 ± 1.3), *Acidothiobacillus* (2.4 ± 0.3), *Sulfuricurvum* (2.5 ± 1.3), and *Sulfurospirillum* (3.1 ± 1.0). *Shewanella* and *Sulfurospirillum* are iron reducing bacteria (FeRB) that may induce (64) or inhibit (65) pipe corrosion by outcompeting sulfur reducing bacteria (SRB). *Acidothiobacillus* and *Sulfuricurvum* are believed to accelerate corrosion through iron or sulfur oxidation, respectively (66, 67). Genera that decreased on average were *Thiobacillus* (log2 fold change = −2.1 ± 3.6), *Desulfosporosinus* (−1.6 ± 2.8), *Geothermobacter* (−1.1 ± 2.3), and *Desulfobulbus* (−1.0 ± 2.1), but results were less consistent. For instance, *Desulfosporosinus* ASVs decreased eightfold in response to all three inhibitors in Set 2 (log2 fold change = −3.0 ± 0) but were only detected in zinc phosphate-appended microcosms in Set 3, where they increased sixfold (2.6). In contrast, *Geothermobacter* ASVs decreased in response to all three inhibitors in Set 3 (log2 fold change = −2.0 ± 1.8) but increased only in the zinc phosphate-appended microcosms in Sets 1 (0.52) and 2 (2.2 ± 0.55). *Geothermobacter* and *Thiobacilllus* are FeRB (68, 69) that also have the potential to outcompete SRB-induced corrosion, while *Desulfobulbus* and *Desulfosporosinus* are SRB linked to steel pipe corrosion (72, 74). *Leptothrix* and *Pseudomonas* ASVs had the most variable responses to treatments. *Leptothrix*, a genus shown to induce corrosion via manganese oxidation (61), were between 300× reduced (log2 fold change = −8.2) and 500× amplified (8.9), both in response to zinc phosphate treatments in Set 2. *Pseudomonas* similarly yielded no consistent response to any treatment. *Pseudomonas* biofilms have been shown to corrode pipes through nitrate reduction (63).

*Pseudomonas* is also associated with waterborne pathogens, along with *Arcobacter*, *Mycobacterium*, and *Legionella* (Table S7; Fig. 4B). On average, *Arcobacter* increased in Set 1 (log2 fold change = 0.91 ± 3.7) and decreased in Set 3 (−2.2 ± 3.8) in response to treatments. *Legionella* also decreased in Set 3 (−1.4 ± 4.2), and *Mycobacterium* was not detected in Set 3, despite being enriched in other untreated source-water experiments (Set 2; 2.8 ± 2.8).

### Relationships between microbial community composition and ARG abundance

Spearman correlation analysis was conducted to determine relationships between identified bacterial genera and the observed abundance of resistance genes as measured with qPCR. Correlation analysis can help determine potential host bacteria harboring ARGs and integrase genes such as *intI*1 (90). The strongest positive correlations with microbial taxa were observed for sulfonamide ARGs *sul*1 and *sul*2 and MRGs including zinc resistance genes *czc*C and *czc*D. The sulfonamide resistance gene *sul*1 was positively correlated with the co-occurrence of *Sediminibacterium*, *Deinococcaceae*, *Ferribacterium*, and *Microscillaceae*. The presence of the sulfonamide resistance gene *sul*2 was also positively associated with *Ferribacterium* and *Microscillaceae*. The *sul*1 gene is associated with a class 1 integron, has been observed to be highly mobile, and is commonly detected in drinking water sources such as lakes and rivers, DWDS, and in tap drinking water (10, 91 – 93). The abundance of *intI*1, *sul*1, *sul*2, and *qacE*Δ1 as quantified with qPCR and their association with several bacteria genera suggest that the selection by corrosion inhibitor addition, especially zinc orthophosphate, may be related to metabolic activity related to zinc homeostasis. Similarly, the results from plating of heterotrophic ARB also indicated increased growth of ARB resistant to SULF in response to zinc orthophosphate addition. The observed relationships between resistance genes and microbial taxa in the samples imply that some of the shifts in observed gene abundance could have resulted from shifts in the abundance and/or types of host bacteria (94).

### Implications for antibiotic resistance and LCR compliance in DWDS

DWDS are important routes for disease transmission and serve as critical control points for the global spread of AMR (27, 95). It is widely documented that disinfectants (free chlorine/chloramines) and metals present in DWDS can select for resistant bacteria and enrich ARGs in tap drinking water (96, 97). The results of this study suggest that utilities using corrosion inhibitors such as zinc orthophosphate may be promoting increased occurrence of bacteria and ARGs in tap drinking water and in DWDS. According to a 2019 American Water Works Association (AWWA) survey, zinc orthophosphate is the most common form of corrosion control with over 38% of utilities reporting its use for LCR compliance (18). The corrosion inhibitor sodium silicate resulted in the least selection potential for ARB and ARGs and may be a preferred option to phosphate-based or metal-containing corrosion inhibitors for controlling antibiotic resistance in DWDS, if the water chemistry of the system allows for selection of any of these corrosion inhibitors.

Implementation of sodium silicate as an alternative corrosion inhibitor may be beneficial for limiting bacterial growth; however, some research indicates that silicates may have reduced ability to mitigate lead release and protect against galvanic corrosion compared to orthophosphates (25). Studies documenting silicate usage have reported higher lead release when compared to orthophosphates for lead and copper control (23, 98 – 100). Others have noted that sodium silicate addition can result in the formation of a passivation layer on the interior surface of pipes, which may help inhibit corrosion (22, 24). Long-term studies should be conducted to determine the impacts on microbial communities from longer durations of exposure to corrosion inhibitors compared to the short-term exposure experiments conducted in this study. Additionally, research that considers the combined effects of disinfectants and corrosion inhibitors would be beneficial for deciphering the complex interactions between chemicals added for water treatment purposes and their potential impacts on antibiotic resistance.

The results presented in this study broaden our understanding of antibiotic resistance selection in drinking water and freshwater ecosystems and stress the importance of monitoring the development and spread of antibiotic resistance in natural and engineered environments (101). Drinking water management practices that minimize the impact of chemicals added for water treatment purposes and mitigate the spread of antibiotic resistance in tap drinking water should be prioritized. For example, corrosion inhibitors that simultaneously reduce dissolved metal concentrations and limit microbial growth in drinking water should be considered for the protection of public and environmental health. Factors such as dissolved metals, metal pipe materials, corrosion inhibitors, and corrosion products that develop in DWDS may be preferentially selected for bacteria harboring resistance to both metals and antibiotics (27, 102, 103). As demonstrated in this study, corrosion inhibitor type and concentration can have a significant impact on the levels of ARB and ARGs as well as on the microbial community structure.

### Conclusions

The results of this study indicate that corrosion inhibitors commonly used in drinking water systems can alter antibiotic resistance profiles and promote the occurrence of ARB, ARGs, and *intI*1 in bacterial communities. Significant increases in absolute and relative abundance of ARB resistant to multiple clinically relevant antibiotics including CIP, SULF, TRIM, and VAN and ARGs including *sul*1, *sul*2, *qac*EΔ1, and *intI*1 were observed in response to zinc orthophosphate addition at concentrations typically found in distribution systems (~1 mg/L). Zinc orthophosphate resulted in the highest selection of ARB and associated ARGs compared to all other conditions tested. This study highlights the need for increased monitoring of antibiotic resistance patterns and microbial communities in engineered systems such as DWDS and in the natural environment.

The microcosm experiments in this study were conducted with a source water for drinking water to capture a broader range of antibiotic resistance selection and reduce the confounding effects of water treatment processes and disinfectant residuals on the initial microbial communities. Interestingly, the type of source water affected the results. Notably, more selection for ARB and ARGs was observed in Sets 2 and 3 (shallow source water collected at recreational beaches) compared to Set 1 (deep source water collected at DWTP intake pipe). It is acknowledged that the site of source water collection can impact bacterial communities in the source water used for the experiments and thus in turn impact the resulting resistance profiles. Additionally, each DWDS likely contains a unique subset of the freshwater microbial communities in its source water. Given the observed impact of corrosion inhibitors on antibiotic resistance, longer-term temporal studies measuring antibiotic resistance in relation to corrosion inhibitors or other environmental factors would be necessary to understand the dynamics of any particular DWDS (101). Also, the impacts due to corrosion inhibitors in DWDS are most relevant in *in situ* pipe biofilms, where organisms are exposed to these inhibitors long term. Both long-term temporal studies in full-scale systems and studies regarding the impacts of corrosion inhibitors, corrosion products, and different pipe materials in laboratory-scale and full-scale systems are required to gain a better understanding of the proliferation of antibiotic resistance in the drinking water environment. Within this study framework, metagenomic analysis of bacterial communities treated with different corrosion inhibitors could provide valuable information regarding selection for antibiotic resistance through analyzing the effects on the microbiome and resistome.

## SUPPLEMENTAL MATERIAL

Supplemental Information msphere.00307-23-s0001.docx Supplemental text, Tables S1-S3, Figures S1-S4, and Tables S8-S9.

Supplemental Tables msphere.00307-23-s0002.xlsx Tables S4-S7.

ASM does not own the copyrights to Supplemental Material that may be linked to, or accessed through, an article. The authors have granted ASM a non-exclusive, world-wide license to publish the Supplemental Material files. Please contact the corresponding author directly for reuse.
