# Supplementary material for: Impact of corrosion inhibitors on antibiotic resistance, metal resistance, and microbial communities in drinking water
Source: mSphere. 2023 Sep 8;8(5):e00307-23. doi: 10.1128/msphere.00307-23 (PMC10597465; doi:10.1128/msphere.00307-23)
Supplement: Supplemental Information — Supplemental text, Tables S1-S3, Figures S1-S4, and Tables S8-S9. [file msphere.00307-23-s0001.docx]

**Supplementary Information**

**Title:** Impact of Corrosion Inhibitor Selection on Antibiotic Resistance, Metal Resistance, and Microbial Communities

**Authors**: Lee K. Kimbell^1^, Stan Kohls^1^, Emily Lou LaMartina^2^, Yin Wang^3^, Ryan J. Newton^2^, Patrick J. McNamara^1^*

**Author Affiliations**:

1. Marquette University, Department of Civil, Construction & Environmental Engineering

2. University of Wisconsin-Milwaukee, School of Freshwater Sciences

3. University of Wisconsin-Milwaukee, Department of Civil and Environmental Engineering

*corresponding author; e-mail: [Patrick.McNamara@marquette.edu](mailto:Patrick.McNamara@marquette.edu)

1637 W Wisconsin Ave, Milwaukee, WI 53233, USA.

**Methods**

**Description of Quantitative PCR Methodology**

All qPCR reactions were performed with 20 µL total reaction volumes with 1x PowerUp SYBR Green Master Mix (Applied Biosystems, USA), F/R primers at a final concentration of 1.0 uM each, and 5 µL of DNA template. DNA templates were diluted to 1:10 for all genes except for the 16S rRNA gene, which was diluted to 1:100 to achieve concentrations within the quantifiable range. Each sample was run in duplicate on qPCR and the results were averaged. The replicates from microcosms were averaged (n = 3 from triplicate experiments, and each of the three values were from the average of duplicate qPCR runs) as previously reported (Kappell et al., 2019). Microcosm samples with results below the quantification limit were reported as the quantification limit. No template controls and standards containing target gene DNA between 10^0^ and 10^7^ copies were performed in duplicate with each qPCR assay. Cycling conditions were conducted as previously described (Kimbell et al., 2021, 2018). Amplification specificity for of target genes was confirmed by melt curves consistent with that of each standard. Amplification efficiency was determined by the resulting standard curve and was considered acceptable between 0.9 and 1.1. Reactions were performed using a LightCycler 96 (Roche Molecular Systems Inc., USA).

**Table S1**. Primers, annealing temperatures, amplification efficiencies, and R^2^ values for qPCR analysis of target genes

| Gene | Annealing Temperature (°C) | Forward Primer & Reverse Primer | Amplicon Size (bp) | Efficiency Average (%) | R^2^ | Reference |
| --- | --- | --- | --- | --- | --- | --- |
| 16S rRNA | 60 | F- (5'-CCTACGGGAGGCAGCAG-3') R- (5'-ATTACCGCGGCTGCTGG-3') | 202 | 98% | 0.98-1.0 | (Muyzer et al., 1993) |
| *bla*_TEM_ | 60 | F- (5'-GCKGCCAACTTACTTCTGACAACG-3') R- (5'-CTTTATCCGCCTCCATCCAGTCTA-3') | 257 | 92% | 0.99-1.0 | (Marti et al., 2013) |
| *cop*A | 63 | F- (5'-ATGTGGAACSARATGCGKATGA-3') R- (5'-AGYTTCAGGCCSGGAATACG-3') | 193 | 90% | 0.98-0.99 | (Roosa et al., 2014) |
| *czc*C | 62 | F- (5’-AGCCGYCAGTATCCGGATCTGAC-3’)  R- (5’-GTGGTCGCCGCCTGATAGGT-3’) | 418 | 90% | 0.98-1.0 | (Roosa et al., 2014) |
| *czc*D | 55 | F- (5'-TCATCGCCGGTGCGATCATCAT-3') R- (5'-TGTCATTCACGACATGAACC-3') | 272 | 90% | 0.98-0.99 | (Roosa et al., 2014) |
| *intI*1 | 60 | F- (5'-CCTCCCGCACGATGATC-3') R- (5'-TCCACGCATCGTCAGGC-3') | 280 | 95% | 0.98-1.0 | (Goldstein et al., 2001) |
| *sul*1 | 60 | F- (5'-CCGTTGGCCTTCCTGTAAAG-3') R- (5'-TTGCCGATCGCGTGAAGT-3') | 67 | 92% | 0.99-1.0 | (Burch et al., 2013) |
| *sul2* | 60 | F- (5'- TCCGATGGAGGCCGGTATCTGG-3') R- (5'- CGGGAATGCCATCTGCCTTGAG-3') | 191 | 90% | 0.98-1.0 | (Muziasari et al., 2014) |
| *qac*EΔ1 | 60 | F- (5’-CCCCTTCCGCCGTTGT-3’)  R- (5’-CGACCAGACTGCATAAGCAACA-3’) | 101 | 95% | 0.98-1.0 | (Zhu et al., 2013) |

**Table S2.** Day 0 Water Quality Characteristics for Microcosm Experiments

| Experiment Set | Microcosm Set 1 | Microcosm Set 2 | Microcosm Set 3 |
| --- | --- | --- | --- |
| Sample Location | DWTP Intake Pipe | Atwater Beach | Atwater Beach |
| Description | Day 0 | Day 0 | Day 0 |
| pH (standard units) | 8.20 | 8.15 | 8.05 |
| Temp (°F) | 62 | 63 | 50 |
| Silica (mg/L as SiO_2_) | <1 | 2 | 4 |
| H_2_S (mg/L) | <0.1 | <0.1 | <0.1 |
| Chloride (mg/L as Cl) | 20 | 30 | 25 |
| Free Chlorine (mg/L as Cl) | <0.1 | <0.1 | <0.1 |
| Total Chlorine (mg/L as Cl) | <0.1 | <0.1 | <0.1 |
| Dissolved Oxygen (mg/L as O_2_) | 13 | 9 | 13 |
| Ammonia (mg/L) | 0.1 | 0.1 | 0.2 |
| Calcium Hardness (mg/L as CaCO_3_) | 100 | 120 | 120 |
| Total Hardness (mg/L as CaCO_3_) | 180 | 180 | 160 |
| Total Alkalinity (mg/L as CaCO_3_) | 140 | 160 | 140 |
| Phenolphthalein Alkalinity (mg/L) | 0 | 0 | 0 |
| Orthophosphate (mg/L as PO_4_) | <0.05 | 0.05 | 0.25 |
| DOC (mg/L as C) | 2.57 | 2.80 | 1.85 |

**Notes:** DOC = dissolved organic carbon, mg/L = milligrams per liter, DWTP = drinking water treatment plant, CaCO_3_ = calcium carbonate, C = carbon, °F = degrees Fahrenheit

**Table S3.** Day 3 and 7 Water Quality for Microcosm Experiments

| **Sample Name** | **Day** | **Initial Dose** | **pH (S.U.)** | **PO4 (mg/L)** | **Silica (mg/L)** | **DOC (mg/L)** |
| --- | --- | --- | --- | --- | --- | --- |
| Control (Set 1) | 3 | NA | 7.92-8.22 | 0.01-0.05 | <1 | 2.72-2.87 |
| Sodium Silicate | 3 | 10 mg/L | 8.01-8.33 | 0.02-0.04 | 10.0-12.0 | 1.69-3.43 |
| Sodium Orthophosphate | 3 | 1 mg/L | 8.12-8.17 | 0.99-1.0 | <1 | 2.58-2.74 |
| Zinc Orthophosphate | 3 | 1 mg/L | 8.22-8.26 | 0.72-1.15 | <1 | 2.59-2.71 |
| Control (Set 1) | 7 | NA | 8.16-8.26 | 0.01-0.02 | <1 | 2.16-2.92 |
| Sodium Silicate | 7 | 10 mg/L | 8.13-8.33 | 0.02-0.03 | 10.0-12.0 | 1.76-2.83 |
| Sodium Orthophosphate | 7 | 1 mg/L | 8.16-8.22 | 0.99-1.11 | <1 | 2.79-2.99 |
| Zinc Orthophosphate | 7 | 1 mg/L | 8.18-8.21 | 0.20-0.83 | <1 | 2.89-3.04 |
| Control (Set 2) | 3 | NA | 7.92-8.22 | 0.05-0.07 | 2 | 2.83-2.99 |
| Sodium Silicate | 3 | 10 mg/L | 8.01-8.30 | 0.08-0.11 | 10.0-12.0 | 2.65-2.89 |
| Sodium Orthophosphate | 3 | 1 mg/L | 8.14-8.17 | 0.7-1.25 | 2 | 2.70-3.18 |
| Zinc Orthophosphate | 3 | 1 mg/L | 8.22-8.26 | 0.7-0.80 | 2 | 2.06-2.40 |
| Control (Set 2) | 7 | NA | 7.86-8.06 | 0.1-0.13 | 2 | 2.05-2.33 |
| Sodium Silicate | 7 | 10 mg/L | 7.81-8.05 | 0.13-0.14 | 10.0-12.0 | 1.97-2.11 |
| Sodium Orthophosphate | 7 | 1 mg/L | 7.62-7.76 | 0.84-1.05 | 2 | 1.96-2.14 |
| Zinc Orthophosphate | 7 | 1 mg/L | 7.8-8.14 | 0.84-1.30 | 2 | 2.17-2.29 |
| Control - Set 3 | 3 | NA | 7.80-7.93 | 0.16-0.22 | 4 | 1.85-2.12 |
| Sodium Silicate | 3 | 100 mg/L | 8.16-8.26 | 0.23-0.27 | 95-100 | 1.81-8.88 |
| Sodium Orthophosphate | 3 | 10 mg/L | 7.65-7.97 | 10.0-13.0 | 4 | 1.88-2.00 |
| Zinc Orthophosphate | 3 | 10 mg/L | 7.79-8.03 | 10.0-14.0 | 4 | 1.95-2.00 |
| Control (Set 3) | 7 | NA | 7.69-7.82 | 0.24-0.33 | 4 | 1.76-1.89 |
| Sodium Silicate | 7 | 100 mg/L | 8.5-8.62 | 0.17-0.36 | 95-100 | 1.79-1.89 |
| Sodium Orthophosphate | 7 | 10 mg/L | 8.06-8.2 | 10.3-12.5 | 4 | 1.83-1.90 |
| Zinc Orthophosphate | 7 | 10 mg/L | 7.98-8.15 | 10.5-12.5 | 4 | 1.89-1.95 |

**Notes:**  DOC = dissolved organic carbon, mg/L = milligrams per liter, NA = not applicable, PO_4_ = orthophosphate

**Figure S1.** Relative abundance of heterotrophic plate counts observed on Day 3 for microcosm experiments. The average relative abundance of antibiotic resistant bacteria (ARB) based on direct plating of from microcosms containing different types and concentrations of corrosion inhibitors. The relative abundance of ARB is shown on the y-axis. The type of antibiotic is denoted on the x-axis. Corrosion inhibitor type is indicated by color. Different experimental conditions are plotted on different graphs including Set 1 – Normal concentration (1X) of CI’s, lake water collected from DWTP (A - top), Set 2 – Normal concentration (1X) of CI’s, lake water collected at beach (B - middle), and Set 3 – High concentration (10X) of CI’s, lake water collected at beach (C - bottom).

**Figure S2.** Relative abundance of heterotrophic plate counts observed on Day 7 for microcosm experiments. The average relative abundance of antibiotic resistant bacteria (ARB) based on direct plating of from microcosms containing different types and concentrations of corrosion inhibitors. The relative abundance of ARB is shown on the y-axis. The type of antibiotic is denoted on the x-axis. Corrosion inhibitor type is indicated by color. Different experimental conditions are plotted on different graphs including Set 1 – Normal concentration (1X) of CI’s, Drinking water collected from DWTP (top), Set 2 – Normal concentration (1X) of CI’s, surface water collected at beach (middle), and Set 3 – High concentration (10X) of CI’s, surface water collected at beach (bottom).

**Table S8.** Summary of differences of absolute abundance (Log10 scale) of total heterotrophic bacteria (R2A) and antibiotic resistant bacteria (ARB) measurements obtained from direct plating onto R2A-media with antibiotics. Values represent the different between the absolute abundance of untreated controls and the average of triplicate treatment reactors for each condition tested.

| **Treatment Type** | **Conc. (mg/L)** | **Source Water** | **Day** | **R2A** | **AMP** | **CIP** | **RIF** | **SULF** | **TET** | **TRIM** | **VAN** |
| --- | --- | --- | --- | --- | --- | --- | --- | --- | --- | --- | --- |
| SS | 10 | SW | 3 | -0.237 | -0.130 | 0.174 | -0.206 | -0.046 | -0.239 | NA | NA |
| NaPO4 | 1 | SW | 3 | -0.370 | 0.097 | 0.291 | -0.082 | -0.247 | -0.653 | NA | NA |
| ZnPO4 | 1 | SW | 3 | 0.011 | 0.039 | 0.125 | 0.266 | -0.062 | -0.349 | NA | NA |
| SS | 10 | SW | 7 | -0.210 | -0.143 | -0.227 | 0.107 | 0.087 | 0.593 | NA | NA |
| NaPO4 | 1 | SW | 7 | -0.763 | 0.097 | -0.080 | 0.130 | 0.710 | 1.393 | NA | NA |
| ZnPO4 | 1 | SW | 7 | -0.280 | -0.003 | 0.000 | -0.157 | 0.127 | -0.333 | NA | NA |
|  | | | AVG | -0.308 | -0.007 | 0.040 | 0.010 | 0.115 | 0.093 | NA | NA |
|  |  |  | SD | 0.340 | 0.565 | 0.423 | 0.352 | 0.820 | 0.822 | NA | NA |
| SS | 10 | SW | 3 | 0.284 | 0.069 | 0.110 | 0.252 | 0.037 | -0.564 | 0.064 | 0.078 |
| NaPO4 | 1 | SW | 3 | **0.554** | 0.527 | 0.484 | **1.082** | **0.970** | -0.292 | **0.559** | **0.810** |
| ZnPO4 | 1 | SW | 3 | **0.771** | **0.979** | **1.080** | **1.093** | **1.449** | **0.858** | **0.713** | **1.369** |
| SS | 10 | SW | 7 | 0.439 | 0.124 | 0.420 | 0.308 | 0.062 | 0.386 | 0.161 | 0.270 |
| NaPO4 | 1 | SW | 7 | 0.292 | 0.269 | 0.442 | 0.172 | 0.216 | 0.332 | 0.179 | 0.268 |
| ZnPO4 | 1 | SW | 7 | 0.663 | 0.774 | **0.857** | 0.722 | **1.069** | 0.572 | **0.950** | **1.290** |
|  | | | AVG | 0.501 | 0.457 | 0.565 | 0.605 | 0.634 | 0.215 | 0.438 | 0.681 |
|  |  |  | SD | 0.291 | 0.475 | 0.457 | 0.542 | 0.629 | 0.569 | 0.363 | 0.570 |
| SS | 100 | SW | 3 | 0.425 | 0.261 | 0.296 | **1.719** | **0.732** | -0.713 | 0.704 | 0.800 |
| NaPO4 | 10 | SW | 3 | 0.585 | 0.706 | 0.481 | 0.483 | **1.087** | -0.016 | 0.619 | 1.000 |
| ZnPO4 | 10 | SW | 3 | 0.697 | 0.383 | 0.133 | 0.683 | **0.999** | 0.593 | 0.927 | **1.429** |
| SS | 100 | SW | 7 | 0.654 | **0.462** | **0.727** | **1.667** | 0.486 | 0.235 | 0.923 | **1.243** |
| NaPO4 | 10 | SW | 7 | 0.020 | 0.126 | **0.458** | -0.013 | 0.214 | -0.167 | 0.412 | **0.438** |
| ZnPO4 | 10 | SW | 7 | **0.962** | **0.958** | **0.979** | **1.222** | **1.256** | **1.535** | 0.956 | **2.000** |
|  | | | AVG | 0.557 | 0.483 | 0.512 | 0.960 | 0.796 | 0.245 | 0.757 | 1.152 |
|  |  |  | SD | 0.468 | 0.453 | 0.345 | 0.684 | 0.421 | 0.787 | 0.351 | 0.602 |

**Notes**: NA = not analyzed, SW = surface water, SS = sodium silicate, ZnPO4 = zinc orthophosphate, NaPO4 = sodium orthophosphate, mg/L = milligrams per liter, AVG = average of absolute abundance measurements, SD = standard deviation of absolute abundance measurements. Yellow highlight indicates any change in relative abundance that was greater than the observed standard deviation. Bold values indicate each result that was statistically different compared to the untreated control (all p values < 0.05).

**Figure S3**. Relative abundance of ARGs, MRGs, and *intI*1 on Day 3 of microcosm experiments as determined by qPCR. The average relative abundance is based on triplicate measurements from microcosms containing different types and concentrations of corrosion inhibitors. The relative abundance of each target gene is shown on the y-axis. The type of gene is denoted on the x-axis. Treatment type is indicated by color. Different experimental conditions are plotted on different graphs including Set 1 – Normal concentration (1X) of CI’s, lake water collected from DWTP (top), Set 2 – Normal concentration (1X) of CI’s, lake water collected at beach (middle), and Set 3 – High concentration (10X) of CI’s, lake water collected at beach (bottom).

**Figure S4**. Relative abundance of ARGs, MRGs, and *intI*1 on Day 7 of microcosm experiments as determined by qPCR. The average relative abundance is based on triplicate measurements from microcosms containing different types and concentrations of corrosion inhibitors. The relative abundance of each target gene is shown on the y-axis. The type of gene is denoted on the x-axis. Corrosion inhibitor type is indicated by color. Different experimental conditions are plotted on different graphs including Set 1 – Normal concentration (1X) of CI’s, lake water collected from DWTP (top), Set 2 – Normal concentration (1X) of CI’s, lake water collected at beach (middle), and Set 3 – High concentration (10X) of CI’s, lake water collected at beach (bottom).

**Table S9.** Summary of differences of absolute abundance (Log10 scale) of total bacterial biomass (16S rRNA), antibiotic resistance genes (ARGs), and metal resistance genes (MRGs) measurements obtained from qPCR. Values represent the difference between the average (n=3) absolute abundance of untreated controls and the average absolute abundance of treatment reactors for each condition tested (n=3).

|  |  |  |  | **Average Log_10_ Difference in Absolute Abundance for Target Genes with qPCR (Treatment - Control)** | | | | | | | | |
| --- | --- | --- | --- | --- | --- | --- | --- | --- | --- | --- | --- | --- |
| **Type** | **Conc. (mg/L)** | **Source Water** | **Day** | **16S rRNA** | ***bla*TEM** | ***czc*C** | ***czc*D** | ***cop*A** | ***intI*1** | ***sul*1** | ***sul*2** | ***qac*EΔ1** |
| SS | 10 | SW | 3 | 0.050 | -0.028 | 0.385 | 0.160 | 0.000 | -0.264 | -0.007 | -0.52 | -0.206 |
| NaPO4 | 1 | SW | 3 | **0.541** | 0.448 | 0.278 | **0.429** | -0.045 | 0.279 | 0.13 | 0 | 0.274 |
| ZnPO4 | 1 | SW | 3 | **0.528** | 0.306 | 0.312 | **0.397** | -0.121 | **0.414** | 0.167 | -0.867 | 0.355 |
| SS | 10 | SW | 7 | 0.386 | -0.147 | 0.066 | 0.166 | 0.229 | 0.381 | -0.083 | -0.391 | 0.425 |
| NaPO4 | 1 | SW | 7 | 0.226 | 0.051 | -0.203 | -0.104 | 0.029 | -0.024 | -0.353 | -0.396 | 0.017 |
| ZnPO4 | 1 | SW | 7 | 0.122 | 0.017 | -0.038 | -0.034 | 0.000 | 0.357 | -0.412 | 0 | 0.426 |
|  | | | AVG | 0.309 | 0.081 | 0.088 | 0.169 | 0.002 | 0.190 | -0.078 | -0.479 | 0.215 |
|  |  |  | SD | 0.263 | 0.280 | 0.234 | 0.256 | 0.192 | 0.357 | 0.266 | 0.269 | 0.345 |
| SS | 10 | SW | 3 | 0.248 | -0.021 | 0.185 | 0.220 | 0.125 | 0.091 | 0.125 | -0.077 | 0.226 |
| NaPO4 | 1 | SW | 3 | 0.256 | -0.349 | 0.040 | 0.109 | -0.103 | 0.178 | 0.292 | 0.144 | 0.193 |
| ZnPO4 | 1 | SW | 3 | 0.120 | -0.216 | -0.152 | -0.142 | -0.172 | 0.655 | **1.083** | 0.224 | **1.198** |
| SS | 10 | SW | 7 | 0.242 | 0.288 | 0.158 | 0.193 | 0.076 | 0.179 | 0.087 | -0.045 | 0.303 |
| NaPO4 | 1 | SW | 7 | 0.277 | 0.085 | 0.104 | 0.194 | 0.129 | 0.216 | 0.484 | -0.077 | 0.381 |
| ZnPO4 | 1 | SW | 7 | 0.468 | 0.063 | 0.509 | 0.490 | 0.334 | **1.185** | **0.889** | **0.817** | **1.452** |
|  | | | AVG | 0.268 | -0.005 | 0.141 | 0.177 | 0.065 | 0.417 | 0.493 | 0.134 | 0.626 |
|  |  |  | SD | 0.195 | 0.247 | 0.328 | 0.298 | 0.225 | 0.445 | 0.456 | 0.371 | 0.588 |
| SS | 100 | SW | 3 | -1.063 | **-0.464** | **-0.650** | -0.265 | **-0.795** | **-0.523** | **-0.969** | **-0.636** | **-0.612** |
| NaPO4 | 10 | SW | 3 | -0.012 | **-0.218** | 0.062 | 0.087 | **-0.330** | -0.008 | 0.079 | -0.143 | -0.136 |
| ZnPO4 | 10 | SW | 3 | 0.182 | -0.195 | 0.138 | 0.023 | **-0.329** | **0.765** | **1.182** | **0.555** | **1.224** |
| SS | 100 | SW | 7 | **-1.502** | **-0.767** | **-0.856** | **-0.648** | -0.099 | **-0.675** | **-0.92** | **-0.206** | **-0.771** |
| NaPO4 | 10 | SW | 7 | 0.089 | **-0.420** | -0.129 | 0.040 | 0.252 | 0.155 | 0.478 | 0.120 | 0.005 |
| ZnPO4 | 10 | SW | 7 | 0.506 | -0.359 | -0.170 | -0.002 | 0.267 | **1.493** | **1.988** | 1.005 | **1.829** |
|  | | | AVG | -0.300 | -0.404 | -0.268 | -0.127 | -0.172 | 0.201 | 0.306 | -0.041 | 0.256 |
|  |  |  | SD | 0.749 | 0.218 | 0.407 | 0.274 | 0.401 | 0.772 | 1.108 | 0.594 | 0.991 |

**Notes**: SW = surface water, SS = sodium silicate, ZnPO4 = zinc orthophosphate, NaPO4 = sodium orthophosphate, mg/L = milligrams per liter, AVG = average of absolute abundance measurements, SD = standard deviation of absolute abundance measurements. Yellow highlight indicates any change in relative abundance that was greater than the observed standard deviation. Bold values indicate each result that was statistically different compared to the untreated control (all p values < 0.05).

**References**

Burch, T.R., Sadowsky, M.J., LaPara, T.M., 2013. Air-drying beds eliminate antibiotic resistance genes and class 1 integrons in residual municipal wastewater solids. Environ. Sci. Technol. 9965–9971.

Goldstein, C., Lee, M.D., Sanchez, S., Phillips, B., Register, B., Grady, M., Liebert, C., Summers, A.O., White, D.G., Maurer, J.J., Hudson, C., 2001. Incidence of Class 1 and 2 Integrases in Clinical and Commensal Bacteria from Livestock , Companion Animals , and Exotics. Antimicrob. Agents Chemother. 45, 723–726. https://doi.org/10.1128/AAC.45.3.723

Kappell, A.D., Harrison, K.R., McNamara, P.J., 2019. Effects of zinc orthophosphate on the antibiotic resistant bacterial community of a source water used for drinking water treatment. Environ. Sci. Water Res. Technol. 5, 1523–1534. https://doi.org/10.1039/C9EW00374F

Kimbell, L.K., Kappell, A.D., McNamara, P.J., 2018. Effect of pyrolysis on the removal of antibiotic resistance genes and class I integrons from municipal wastewater biosolids. Environ. Sci. Water Res. Technol. 4, 1807–1818. https://doi.org/10.1039/C8EW00141C

Kimbell, L.K., LaMartina, E. Lou, Kappell, A.D., Huo, J., Wang, Y., Newton, R.J., McNamara, P.J., 2021. Cast iron drinking water pipe biofilms support diverse microbial communities containing antibiotic resistance genes, metal resistance genes, and class 1 integrons. Environ. Sci. Water Res. Technol. https://doi.org/10.1039/d0ew01059f

Marti, E., Jofre, J., Balcazar, J.L., 2013. Prevalence of Antibiotic Resistance Genes and Bacterial Community Composition in a River Influenced by a Wastewater Treatment Plant. PLoS One 8, e78906. https://doi.org/10.1371/journal.pone.0078906

Muyzer, G., Waal, E. de, Uitierlinden, A.G., 1993. Profiling of complex microbial populations by denaturing gradient gel electrophoresis analysis of polymerase chain reaction-amplified genes coding for 16S rRNA. Appl. Environmantal Microbiol. 59, 695–700.

Muziasari, W.I., Managaki, S., Pärnänen, K., Karkman, A., Lyra, C., Tamminen, M., Suzuki, S., Virta, M., 2014. Sulphonamide and trimethoprim resistance genes persist in sediments at Baltic Sea aquaculture farms but are not detected in the surrounding environment. PLoS One 9, 1–7. https://doi.org/10.1371/journal.pone.0092702

Roosa, S., Wattiez, R., Prygiel, E., Lesven, L., Billon, G., Gillan, D.C., 2014. Bacterial metal resistance genes and metal bioavailability in contaminated sediments. Environ. Pollut. 189, 143–151. https://doi.org/10.1016/j.envpol.2014.02.031

Zhu, Y.-G., Johnson, T.A., Su, J.-Q., Qiao, M., Guo, G.-X., Stedtfeld, R.D., Hashsham, S.A., Tiedje, J.M., 2013. Diverse and abundant antibiotic resistance genes in Chinese swine farms. Proc. Natl. Acad. Sci. 110. https://doi.org/10.1073/pnas.1222743110
